# Supplementary material for: The Development and Synthesis of a CdZnS @Metal–Organic Framework ZIF-8 for the Highly Efficient Photocatalytic Degradation of Organic Dyes
Source: Molecules. 2023 Dec 2;28(23):7904. doi: 10.3390/molecules28237904 (PMC10708100; doi:10.3390/molecules28237904)
Supplement: Supplementary file 1 [file molecules-28-07904-s001.zip › molecules-2550013-supplementary.pdf]

Support Information

The Development and Synthesis of a CdZnS @Metal–Organic Framework ZIF-8 for the Highly Efficient Photocatalytic Degradation of Organic Dyes

Liu Hong, Jiaming Cao, Wenlong Zhang, Tao Jiang, Guohao Pan and Yun Wu *

School of Energy Materials and Chemical Engineering, Hefei University, Hefei 230601, China; hl18324702376@163.com (L.H.); cjm15155934415@163.com (J.C.); zwnl1628069773@163.com (W.Z.); m158554288245@163.com (T.J.); guohaopan@163.com (G.P.)

* Correspondence: cloudless@hfuu.edu.cn; Tel.: +86-13956024212

1. Characterizations

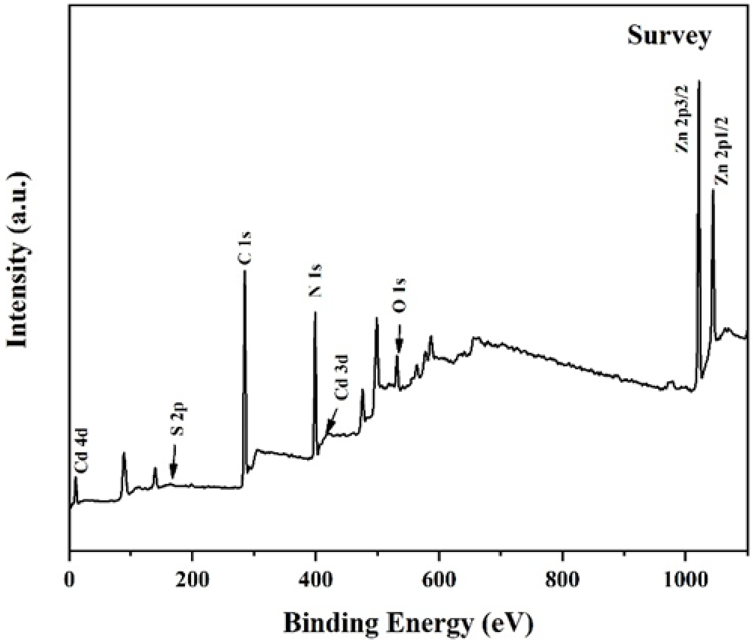

Figure S1. XPS full spectrum of composite materials.

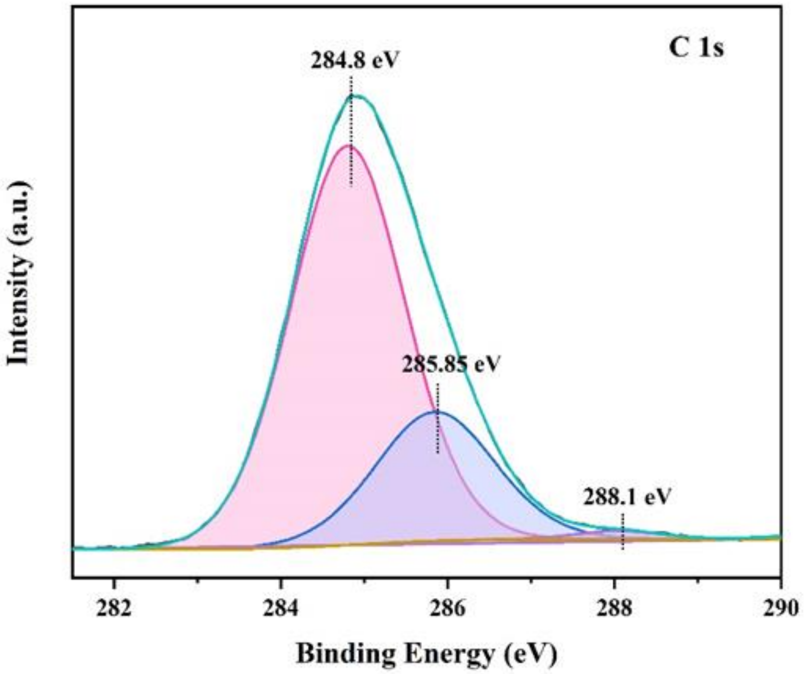

Figure S2. XPS spectrum of C1s.

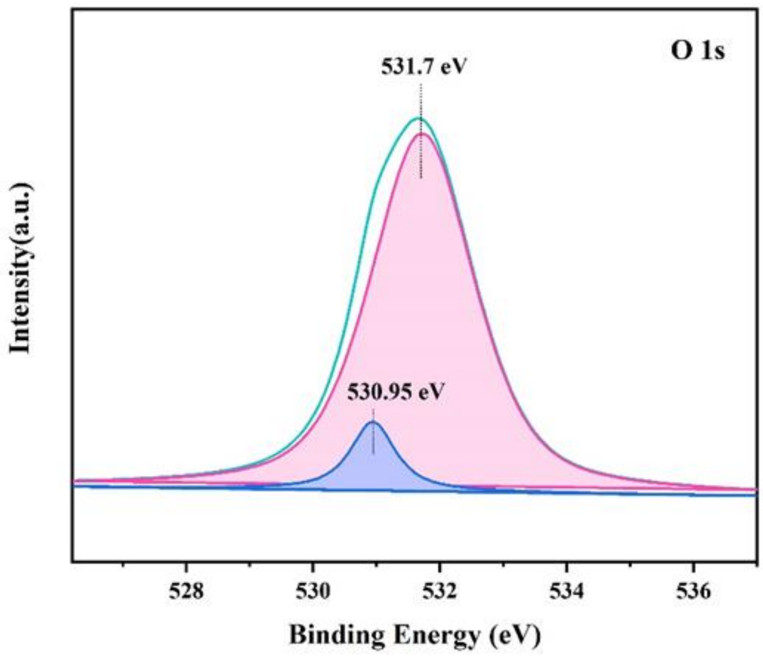

Figure S3. XPS spectrum of O1s.

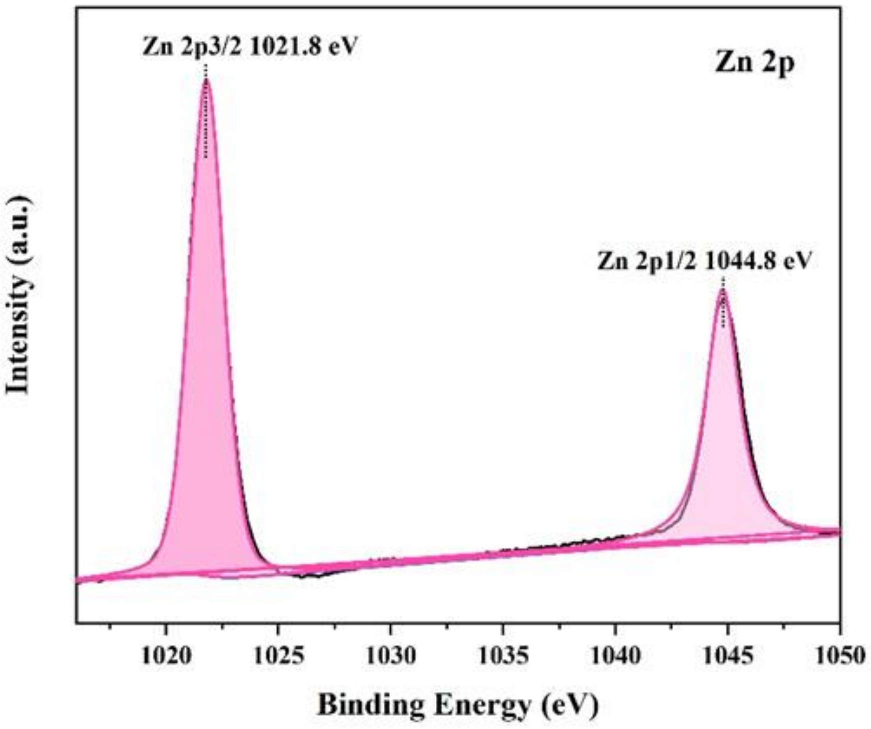

Figure S4. XPS spectrum of Zn 2p.

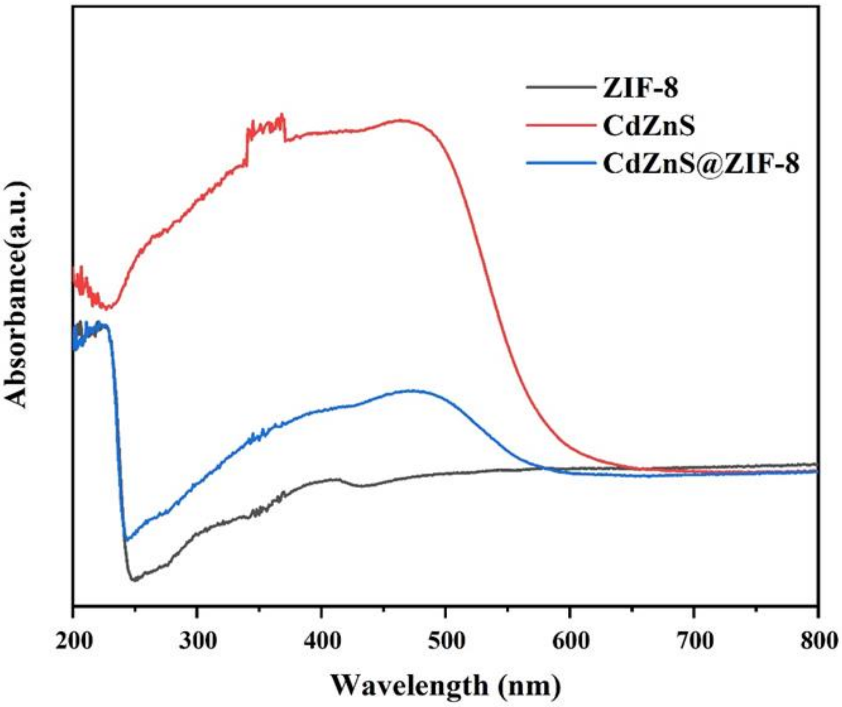

Figure S5. Ultraviolet-visible-near-infrared diffuse reflectance spectra of ZIF-8, CdZnS and CdZnS@ZIF-8.

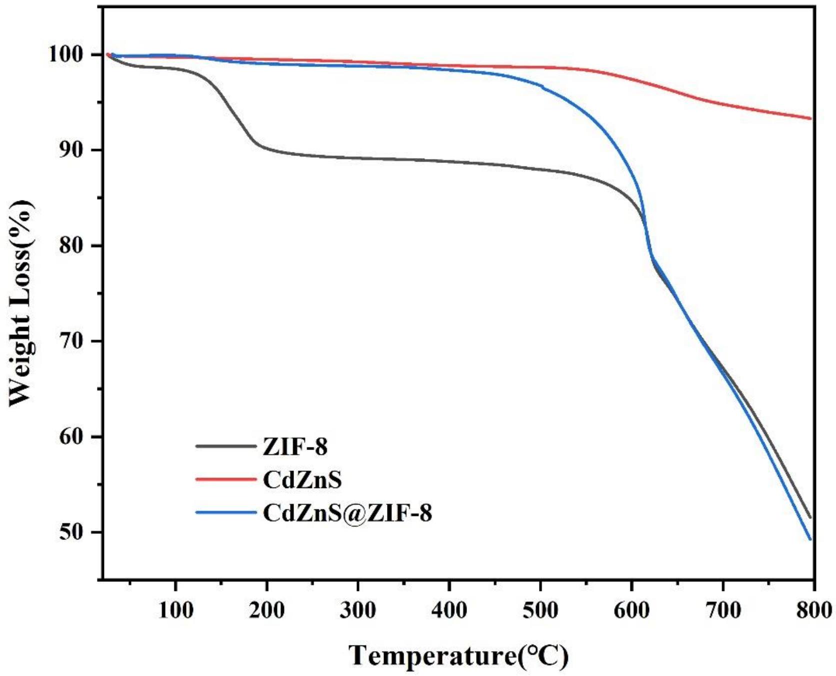

Figure S6. Thermogravimetric of ZIF-8, CdZnS and CdZnS@ZIF-8.

Table S1. Specific surface area and pore size of ZIF-8,CdZnS and CdZnS@ZIF-8.

**Material**	**S_{BET} (m^2g^{-1})**	**Pore Size(nm)**
ZIF-8	1836.352	3.820
CdZnS	12.662	43.140
CdZnS@ZIF-8	1151.684	2.942
